# Molecular mechanisms and therapeutic potential of lithium in Alzheimer’s disease: repurposing an old class of drugs

**DOI:** 10.3389/fphar.2024.1408462

**Published:** 2024-07-11

**Authors:** Yanxin Shen, Meng Zhao, Panpan Zhao, Lingjie Meng, Yan Zhang, Guimei Zhang, Yezi Taishi, Li Sun

**Affiliations:** ^1^ Department of Neurology and Neuroscience Center, The First Hospital of Jilin University, Jilin University, Changchun, Jilin Province, China; ^2^ Cognitive Center, Department of Neurology, The First Hospital of Jilin University, Jilin University, Changchun, Jilin Province, China; ^3^ Department of Cadre Ward, The First Hospital of Jilin University, Jilin University, Changchun, Jilin Province, China

**Keywords:** Alzheimer’s disease, clinical trial, glycogen synthase kinase-3, lithium, neuroprotection, side effect, treatment

## Abstract

Alzheimer’s disease (AD) is a progressive neurodegenerative disorder characterized by cognitive decline and memory loss. Despite advances in understanding the pathophysiological mechanisms of AD, effective treatments remain scarce. Lithium salts, recognized as mood stabilizers in bipolar disorder, have been extensively studied for their neuroprotective effects. Several studies indicate that lithium may be a disease-modifying agent in the treatment of AD. Lithium’s neuroprotective properties in AD by acting on multiple neuropathological targets, such as reducing amyloid deposition and tau phosphorylation, enhancing autophagy, neurogenesis, and synaptic plasticity, regulating cholinergic and glucose metabolism, inhibiting neuroinflammation, oxidative stress, and apoptosis, while preserving mitochondrial function. Clinical trials have demonstrated that lithium therapy can improve cognitive function in patients with AD. In particular, meta-analyses have shown that lithium may be a more effective and safer treatment than the recently FDA-approved aducanumab for improving cognitive function in patients with AD. The affordability and therapeutic efficacy of lithium have prompted a reassessment of its use. However, the use of lithium may lead to potential side effects and safety issues, which may limit its clinical application. Currently, several new lithium formulations are undergoing clinical trials to improve safety and efficacy. This review focuses on lithium’s mechanism of action in treating AD, highlighting the latest advances in preclinical studies and clinical trials. It also explores the side effects of lithium therapy and coping strategies, offering a potential therapeutic strategy for patients with AD.

## 1 Introduction

As the global population grows and ages, the prevalence of dementia is projected to rise from 57.4 million cases in 2019 to 152.8 million by 2050 (GBD, 2019 Dementia Forecasting Collaborators, 2022). Dementia imposes a heavy economic burden on societies worldwide. The World Alzheimer Report 2019 estimates the annual cost of dementia at $1 trillion, a figure projected to double by 2030 (Anon, 2020). Alzheimer’s disease (AD) is the predominant cause of dementia, accounting for approximately 60%–80% of cases (Anon, 2020). Individuals with AD typically experience progressive worsening memory loss, learning disabilities, mental and behavioral changes, and impairments in daily activities (Burns and Iliffe, 2009). Despite extensive research into AD, its pathogenesis remains poorly understood, and no cure has been found. The hallmark neuropathologic features of AD currently include the extracellular deposition of amyloid-beta (Aβ) plaques and intraneuronal neurofibrillary tangles (NFTs) (Bloom, 2014; de Wolf et al., 2020). In addition, the pathophysiology of AD involves the cholinergic neuronal loss, mitochondrial dysfunction, inflammation, oxidative stress, metal ion deposition, gut dysbiosis, abnormal autophagy, and disturbances in calcium homeostasis (Liu et al., 2019; Liu S. et al., 2020). AD is associated with several risk factors, including age, genetics, head trauma, vascular disease, infections, environmental exposures (heavy metals, trace metals), and lifestyle choices (Kivipelto et al., 2018; Huat et al., 2019; Raulin et al., 2022; Bruno et al., 2023). Pharmacological interventions may provide moderate symptomatic relief. Current treatments for AD mainly involve acetylcholinesterase inhibitors, such as donepezil, for mild to severe dementia, and N-methyl-D-aspartic acid receptor antagonists, such as memantine, for moderate to severe dementia (Arvanitakis et al., 2019). In addition, non-pharmacological approaches, including cognitive training, and psychological support, play a vital role in managing AD (Kivipelto et al., 2018; Sikkes et al., 2021). Recently, the U.S. Food and Drug Administration (FDA) has approved two monoclonal antibodies, aducanumab and lecanemab, for global marketing. These antibodies have shown significant success in reducing Aβ levels, but their efficacy in improving cognition remains unsatisfactory (Dhillon, 2021; Larkin, 2023; Wojtunik-Kulesza et al., 2023). This highlights the complexity of AD pathogenesis. The management and treatment of AD often require multi-targeted drugs or combinations of different interventions, as treatment of a single factor is often ineffective in improving symptoms. In conclusion, the treatment of AD still poses significant challenges, and it is crucial to find more optimal therapeutic strategies.

Lithium salts are approved by the FDA for treating manic episodes and bipolar disorder (Goodwin, 2002). It is noteworthy that lithium has the potential to target multiple pathological events in AD, as evidenced by promising results in preclinical and clinical trials. Preclinical studies have shown that lithium can reduce amyloid deposition and tau phosphorylation, regulate autophagy, inflammation, oxidative stress, cholinergic and glucose metabolism, enhance neurogenesis and synaptic plasticity, maintain mitochondrial homeostasis, and improve cognitive function (Fiorentini et al., 2010; Toledo and Inestrosa, 2010; Zhang et al., 2011; Sudduth et al., 2012; Trujillo-Estrada et al., 2013; Wilson et al., 2017; Pan et al., 2018; Wilson et al., 2018; Habib et al., 2019; Liu M. et al., 2020; Wilson et al., 2020; Xiang et al., 2020; Xiang et al., 2021; Gherardelli et al., 2022; Lu et al., 2022; Wiseman et al., 2023). Clinical studies have indicated that lithium therapy can reduce the risk of AD, halt the progression of early-stage AD, and maintain cognitive stability over extended periods (Forlenza et al., 2019; Haussmann et al., 2021). These findings support the effectiveness of lithium as a disease-modifying therapy for AD. Recent meta-analyses have highlighted the effectiveness of lithium in enhancing cognitive function in patients with mild cognitive impairment (MCI) and AD. Specifically, these studies compared lithium to recently FDA-approved or reviewed drugs, such as aducanumab, lecanemab, and donanemab. The results suggest that lithium may be superior in improving cognitive symptoms in AD and may have greater safety at lower doses compared to the other drugs (Terao et al., 2022; Singulani et al., 2024; Terao and Kodama, 2024). In terms of cost, aducanumab is priced at approximately $28,000 per person per year, while lithium costs only about $40 per year (Terao et al., 2022). The efficacy and cost benefits of lithium have prompted a reassessment of this conventional medication. Although the exact mechanism by which lithium acts in AD treatment remains unclear, its inhibition of glycogen synthase kinase-3beta (GSK-3β) and modulation of inositol monophosphatase (IMPase) are thought to be crucial and are the focus of this review (Chalecka-Franaszek and Chuang, 1999; Sarkar and Rubinsztein, 2006). Nevertheless, there are still concerns about the safety and potential side effects of lithium (McKnight et al., 2012). Although studies suggest that lithium use in older patients with AD results in few mild side effects that resolve with discontinuation, potential risks associated with lithium therapy should not be overlooked (Macdonald et al., 2008).

This review explores the mechanism by which lithium acts in the treatment of AD, recent advances in preclinical and clinical trials, and addresses potential side effects and safety concerns of lithium therapy, along with proposed countermeasures. The objective of this review is to highlight the potential of lithium as a treatment for AD and propose a possible therapeutic strategy for patients with AD.

## 2 The history of lithium in medical research

**FIGURE 1:**
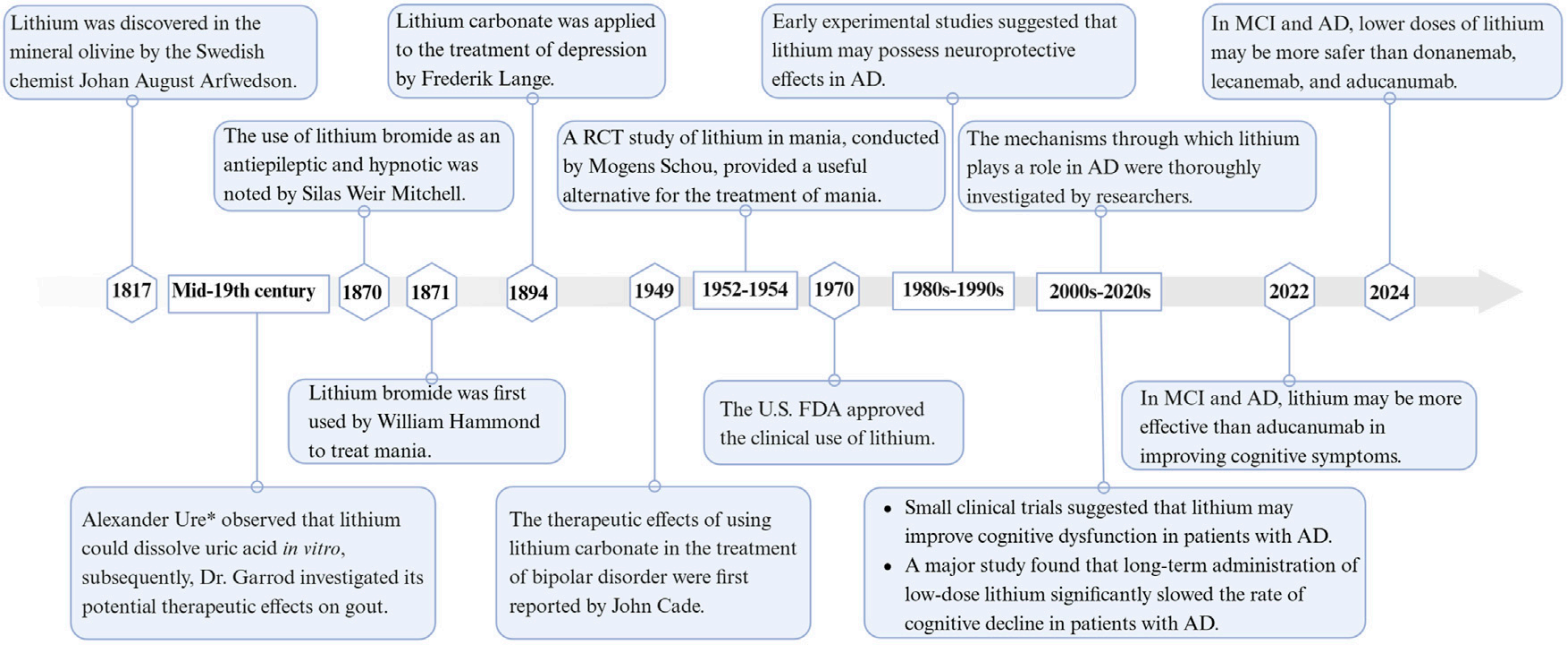
Timeline of lithium in medical research. Created with BioRender.com.

Lithium, the lightest solid element, was discovered in 1817 by the Swedish chemist Johan August Arfwedson in the mineral olivine (Damri et al., 2020). Lithium has been used in the medical field, including psychiatry, for many years, dating back to the mid-19th century (Shorter, 2009). Alexander Ure* first observed that lithium could dissolve uric acid *in vitro*. Later, Dr. Garrod explored its potential therapeutic effects on gout (Anon, 1860). In 1870, Silas Weir Mitchell, a neurologist from Philadelphia, noted that lithium bromide was superior to other bromides as an antiepileptic and hypnotic (Anon, 1870). The following year, Professor William Hammond of New York used lithium bromide for the first time to treat mania. In 1894, Danish psychiatrist Frederik Lange applied lithium carbonate to treat depression (Shorter, 2009). In 1949, psychiatrist John Cade from Melbourne, Australia, used lithium citrate and lithium carbonate to treat manic patients, many of whom responded positively (Cade, 1949). John Cade’s discovery was significant in reintroducing lithium into psychiatric treatment (Bech, 2006). In 1952, Danish psychiatrist Mogens Schou conducted a randomized controlled trial of lithium in mania, influenced by John Cade’s article, and published the study’s results in a British journal in 1954 (Schou et al., 1954). This provided a viable alternative for the treatment of mania. Lithium research in the United States started in the 1960s. By 1970, the U.S. FDA had approved its marketing, making the United States the 50th country to approve lithium (Shorter, 2009). These studies have shown that lithium is significantly effective as a mood stabilizer in bipolar disorder, particularly in preventing manic and depressive relapses. Since 1970, researchers have explored the effects of lithium therapy on cognitive function (Squire et al., 1980; Muñoz-Montaño et al., 1997; Wei et al., 2000). Since 2000, several small clinical trials have evaluated the effects of lithium on cognitive function in patients with AD, as well as investigated the mechanisms of lithium’s action (Forlenza et al., 2011; Matsunaga et al., 2015). Despite inconsistent findings, most studies have supported lithium’s effectiveness in treating AD. Recent systematic reviews and meta-analyses have further confirmed lithium’s effectiveness in treating AD (Terao et al., 2022; Singulani et al., 2024; Terao and Kodama, 2024). Figure 1 illustrates the timeline of lithium research in medicine from its discovery in 1817–2024, with a focus on AD.

**FIGURE 2:**
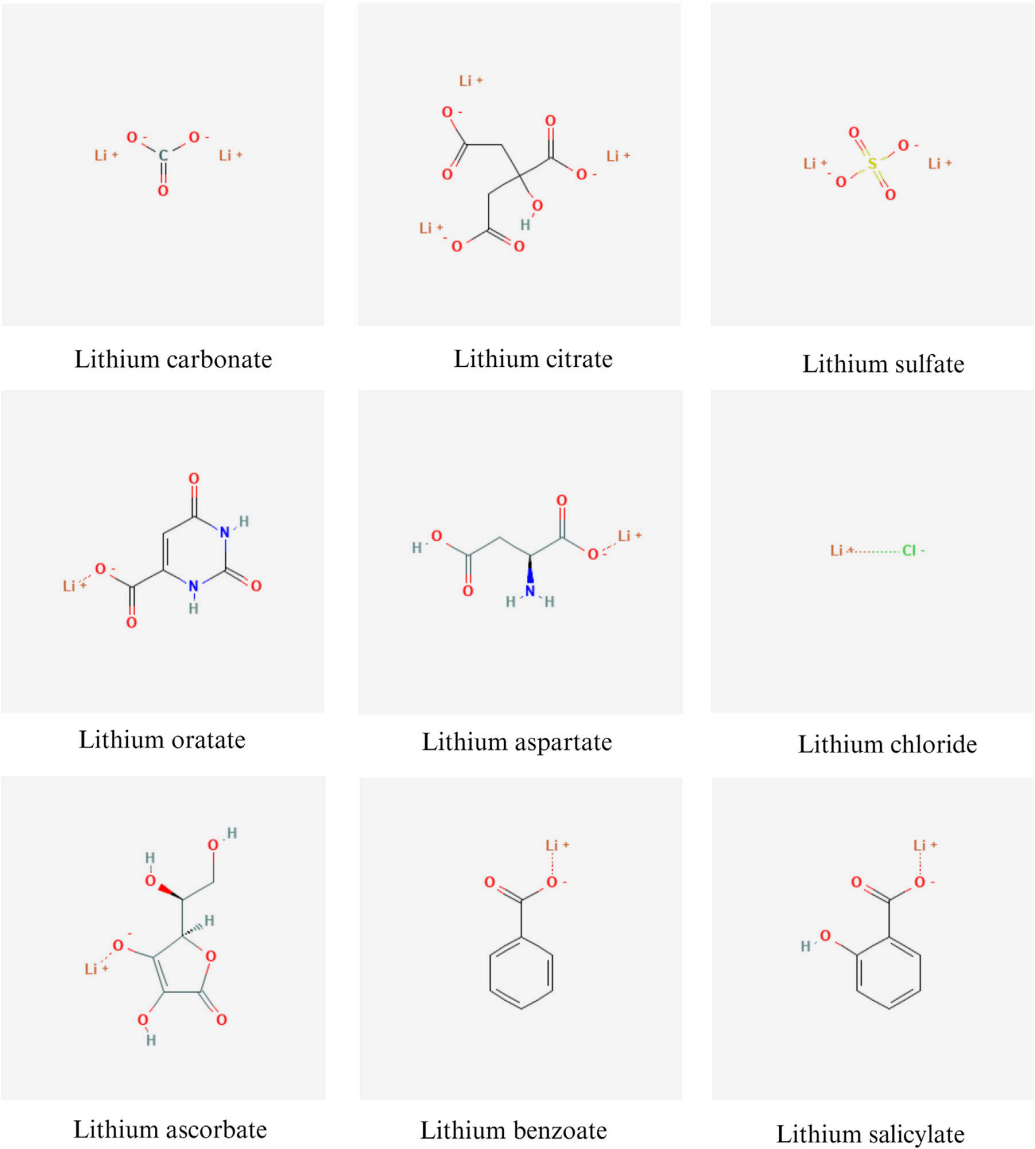
Chemical structures of various lithium salts. Created with BioRender.com. Chemical structures were sourced from the open chemistry database, PubChem, available at https://pubchem.ncbi.nlm.nih.gov/.

Lithium is a metallic element that exhibits monovalent cation properties in chemical reactions and naturally forms salt structures with anions. Psychiatrists commonly prescribe lithium carbonate (Li_2_CO_3_) and lithium citrate (Li_3_C_6_H_5_O_7_) as medications. Alternatives to these include lithium sulfate (Li_2_SO_4_), lithium orotate (C_5_H_3_LiN_2_O_4_), and lithium aspartate (C_4_H_5_Li_2_NO_4_) (Oruch et al., 2014; Pacholko and Bekar, 2021). Figure 2 summarizes the chemical structures of various lithium salts. Different lithium salts may exhibit variations in chemical composition, bioavailability, pharmacodynamic characteristics, and clinical applications. Currently, lithium carbonate and lithium chloride (LiCl) are the primary lithium salts utilized in preclinical studies of AD, as shown in Table 1 (Xiang et al., 2021; Gherardelli et al., 2022). Due to the side effects and toxicity of lithium salts, there is an urgent need to explore non-toxic lithium formulations (Gitlin, 2016; Kakhki and Ahmadi-Soleimani, 2022). Lithium ascorbate, which has low acute and chronic toxicity, has been found to mitigate ischemia-induced brain damage and play a significant neuroprotective role (Torshin et al., 2022). In studies on AD, lithium benzoate and lithium cholesterol sulfate have been shown to improve cognitive and memory functions in animal models through multiple pathways (Hu et al., 2022; Lu et al., 2022). Nanolithium, which utilizes Medesis Pharma’s innovative drug delivery technology (Aonys^®^) to enhance the bioavailability of lithium and reduce its toxicity, has demonstrated potential in preclinical studies with the microdose lithium formulation (NP03) for the treatment of AD (Wilson et al., 2020). Clinical trials of Nanolithium are currently underway for patients with mild to severe AD (ClinicalTrials.gov ID: NCT05423522) (Guilliot et al., 2024). Additionally, AL001 (LISPRO), an ionic co-crystal of lithium salicylate and l-proline designed for targeted brain delivery to enhance efficacy and minimize toxicity, has prevented hippocampal-dependent associative memory decline in AD mouse models (Habib et al., 2019). Clinical trials are currently being conducted on patients with mild to moderate AD (ClinicalTrials.gov ID: NCT05363293). These new lithium formulations are expected to provide a new treatment option for AD. More information on the role of lithium salts in AD can be found in the following Mechanisms section.

**TABLE 1:** Summary of experimental studies on lithium treatment for Alzheimer’s Disease.

Drug	Results	AD models	Lithium level/dosage	References
LiCl	Reducing tau phosphorylation	Primary rat neurons	20 mM for 7 h	Muñoz-Montaño et al. (1997)
LiCl	Reducing tau phosphorylation; enhancing tau binding to microtubules; promoting microtubule assembly	Overexpression of GSK-3β in human NT2N neurons	10 mM for 2 h	Hong et al. (1997)
LiCl	Reducing tau phosphorylation	Primary rat neurons and COS and CHO cells	0–25 mM	Lovestone et al. (1999)
LiCl	Reducing tau mRNA levels; protecting cultured neurons against Aβ toxicity	Primary rat neurons	1.25–7.5 mM for 8 h	Rametti et al. (2008)
LiCl	Reducing tau phosphorylation	Primary rat neurons	10 mM for 3 h	Alvarez et al. (1999)
LiCl	Improving spatial memory; reducing Aβ and P-tau levels	APP/PS1 mice	5 and 17.5 mg/kg by gavage once daily for 2 months	Liu et al. (2020a)
LiCl	Reversing the declined activities of SOD and GSH-Px, the increasing content of MDA and the decreased Nissl bodies; regulating GSK-3β/NRF2/HO-1 pathway	APP/PS1 mice	LiCl 100 mg/kg by gavage once daily for 2 months	Xiang et al. (2020)
LiCl	Improving learning and memory function; reducing senile plaque count and Aβ_1-42_ level; improving the level of α7 nAChR protein; activating Wnt signaling	APP/PS1 mice and primary rat neurons	LiCl 100 mg/kg by gavage once daily for 2 months	Xiang et al. (2021)
LiCl	Increasing brain Aβ clearance, brain microvascular LRP1 expression, and CSF bulk-flow; restoring long-term spatial memory deficits; reducing soluble Aβ levels	APP/PS1 mice	LiCl 300 mg/kg by gavage once daily for 21 days	Pan et al. (2018)
LiCl	Reducing Aβ production and senile plaque formation; improving spatial learning and memory function; attenuating the autophagy activation	APP/PS1 mice	LiCl 0.18 mmol injected intraperitoneally once daily for 3 months	Zhang et al. (2011)
LiCl	Improving spatial memory; reducing Aβ aggregates and Aβ oligomers, astrocytic and microglia activation; preventing changes in presynaptic and postsynaptic marker proteins; activating Wnt signaling	APP/PS1 mice	LiCl 3mequiv.kg^-1^ injected intraperitoneally once daily for 3 months	Toledo and Inestrosa (2010)
LiCl	Inhibiting GSK-3; activating Wnt/β-catenin signaling; stimulating adult hippocampal neurogenesis; improving cognitive functions; reducing Aβ deposition and glia reaction	TgCRND8 mice	Injected intraperitoneally LiCl 0.6 mol/L (10 μL/g of body weight) once daily for 5 weeks	Fiorentini et al. (2010)
LiCl	Reducing IP3R Ca^2+^ and VGCC Ca^2+^ responses in CA1 pyramidal neurons; reducing nNOS and tau phosphorylation; enhancing short term plasticity	3×Tg-AD mice	Diet chow containing 0.4% LiCl (approximately 2 g/kg of Li) for 30 days	Wiseman et al. (2023)
LiCl	Reducing tau phosphorylation; does not alter the Aβ load and rescue working memory deficits	3×Tg-AD mice	LiCl 300 μL of 0.6 mol/L injected intraperitoneally once daily for 4 weeks	Caccamo et al. (2007)
LiCl	Inhibiting GSK-3 activity; reducing tau phosphorylation and the level of aggregated insoluble tau	Transgenic mice overexpressing mutant human tau	Plasma concentration at 24 h post-injection ≈0.2 mEq/L	Noble et al. (2005)
LiCl	Preventing tau hyperphosphorylation and neurofibrillary tangle formation, but pre-formed neurofibrillary tangles do not revert	FTDP-17 tau and GSK-3β overexpressing mice	Preventative effects: 12-month-old mice diet chow containing 1.7 g LiCl/kg for 7.5months; reversion effects: 18-month-old mice diet chow containing 1.7 g LiCl/kg chow for 4 weeks, followed by a diet containing 2.55 g LiCl/kg chow for 2 weeks	Engel et al. (2006)
LiCl	Enhancing soluble tau autophagy clearance; inhibiting GSK-3; attenuating motor disturbance	P301L mice	Diet chow containing 2 g of LiCl per kg for 2 months from 5 months of age followed by 1 g of LiCl per kg chow for another 2 months	Shimada et al. (2012)
Li_2_CO_3_	Stimulating glucose uptake; promoting glycolysis and ATP synthesis; enhancing glycolysis and AMPK activity	APP/PS1 mice and primary rat neurons	Hippocampal neuronal cultures were treated with Li_2_CO_3_ (10 mM); hippocampal slices from APP/PS1 mice were treated with Li_2_CO_3_ (10 mM)	Gherardelli et al. (2022)
Li_2_CO_3_	Rescuing behavioral/memory deficits; preventing neuronal loss; ameliorating axonal/synaptic pathology by reducing abnormal intracellular protein accumulation; modifying the morphology and toxicity of the extracellular Aβ plaques; inducing astrocyte activation	APP/PS1 mice	Diet chow containing Li_2_CO_3_ 1.2 g/kg for 6 months	Trujillo-Estrada et al. (2013)
Li_2_CO_3_	Increasing telomere length in parietal cortex and hippocampus	3×Tg-AD mice	Diet chow containing 1.0 g (Li1) or 2.0 g (Li2) of Li_2_CO_3_/kg for 8 months	Cardillo et al. (2018)
Li_2_CO_3_	Blocking development of NFTs in mutant tau transgenic mice with advanced neurofibrillary fiber pathology	Tgtau30 mice	Diet chow containing 2.4 g of Li_2_CO_3_ per kg for 8 months starting at the age of 3 months (to test for a preventive effect on NFTs formation) or by oral gavage (350 mg per kg of animal weight, dissolved in water) for 1 month starting at the age of 9 months (after development of NFTs)	Leroy et al. (2010)
Li_2_CO_3_	Improving memory function; decreasing exploration activity; increasing the activity of acetylcholinesterase	Scopolamine-induced zebrafish	100 mg/L for 7 days	Zanandrea et al. (2018)
Li_2_CO_3_	Rescuing memory performances but did not modulate ChAT availability and caspase-3 activity	Basal forebrain cholinergic depletion Wistar rats	Diet chow containing of 0.24% Li_2_CO_3_ diet for 30 days	Gelfo et al. (2017)
Li_2_CO_3_	Reducing the levels of IL-1β and TNF-α; reversing the decreased levels of IL-4	Aβ_1-42_ oligomers-induced Wistar rats	Oral treatments with memantine (5 mg/kg), lithium (5 mg/kg), or both drugs in combination for 17days	Budni et al. (2017)
Lithium diet	Reducing tau phosphorylation; altering the neuroinflammatory phenotype and lowering inflammatory response	APPSwDI/NOS2^−/−^mouse model	Diet chow containing of 2 g lithium/kg diet for 8 months	Sudduth et al. (2012)
Lithium benzoate	Inhibiting ROS production; improving mitochondrial function; promoting neurogenesis; improving spatial memory	APP/PS1 mice and primary rat hippocampal neuronal	Injected intraperitoneally once daily, LiBen at 256 mg/kg/day (Li: 14 mg/kg/day) were applied initially for 5 months and the dose was lower to 200 mg/kg/day (Li: 10.9 mg/kg/day) to prevent toxicity for 3 months	Lu et al. (2022)
NP03	Reversing object recognition impairments; protecting against cholinergic bouton loss; reducing soluble and aggregated Aβ_1-42_ in brain and plasma; lowering oxidative stress marker 4-hydroxynonenal; reducing pro-inflammatory mediators	McGill-R-Thy1-APP transgenic rats	40 μg Li/kg; 1 mL/kg body weight on rectal mucosa 5 days per week for 12 weeks	Wilson et al. (2020)
NP03	Decreasing cerebral 4-hydroxynonenal and 3-nitrotyrosine; reducing production of pro-inflammatory cytokines, expression of microglia surface receptor Trem2, and microglial recruitment to Aβ-loaded neurons	McGill-R-Thy1-APP transgenic rats	40 μg Li/kg; 1 mL/kg body weight on rectal mucosa 5 days per week for 8 weeks	Wilson et al. (2018)
NP03	Inhibiting GSK-3β; restoring native β-catenin; reducing BACE1 expression and activity and Aβ levels; reversing memory impairments; rescuing adult hippocampal neurogenesis and synaptic plasticity	McGill-R-Thy1-APP transgenic rats	40 μg Li/kg; 1 mL/kg body weight on rectal mucosa 5 days per week for 8 weeks	Wilson et al. (2017)
LISPRO	Preventing spatial cognitive decline, depression-like behavior, hippocampus-dependent associative memory decline and irritability	APP/PS1 mice	Orally treated with low-dose Li_2_CO_3_, or lithium salicylate at 2.25 mmol lithium/kg/day for 9 months	Habib et al. (2019)

Aβ, amyloid-beta; AD, Alzheimer’s disease; AMPK, adenosine 5-monophosphate (AMP)-activated protein kinase; APP, amyloid precursor protein; ATP, adenosine triphosphate; BACE1, β-site APP, cleaving enzyme 1; ChAT, choline acetyltransferase; CSF, cerebrospinal fluid; GSH-Px, glutathione peroxidase; GSK-3β, glycogen synthase kinase-3β; HO-1, heme oxygenase-1; IL-1β, interleukin-1β; IP3R, inositol 1,4,5-trisphosphate (IP3) receptor; LiCl, lithium chloride; LRP1, low-density lipoprotein receptor-related protein-1; MDA, malondialdehyde; nAChR, neuronal nicotinic acetylcholine receptors; NFTs, neurofibrillary tangles; nNOS, neuronal nitric oxide synthase; NRF2, nuclear factor E2 related factor; PS1, presenilin-1; ROS, reactive oxygen species; SOD, superoxide dismutase; TNF-α, tumor necrosis factor-alpha; VGCC, voltage gated calcium channel; 3×Tg-AD, triple-transgenic AD.

## 3 Potential mechanisms of lithium treatment for Alzheimer's disease

In preclinical research, lithium has been shown to exhibit a variety of neuroprotective properties. Table 1 summarizes the findings of preclinical research on lithium therapy in AD. Lithium exposure in neuronal cultures can reduce total tau and P-tau levels and protect against Aβ toxicity (Hong et al., 1997; Muñoz-Montaño et al., 1997; Alvarez et al., 1999; Lovestone et al., 1999; Rametti et al., 2008). In Aβ-overexpressing AD transgenic mice, lithium therapy has been found to inhibit GSK-3 activity, reduce Aβ deposition and tau phosphorylation, regulate autophagy, inflammation, oxidative stress, cholinergic and glucose metabolism, enhance neurogenesis and synaptic plasticity, maintain mitochondrial homeostasis, and improve cognitive function (Fiorentini et al., 2010; Toledo and Inestrosa, 2010; Zhang et al., 2011; Sudduth et al., 2012; Trujillo-Estrada et al., 2013; Wilson et al., 2017; Pan et al., 2018; Wilson et al., 2018; Habib et al., 2019; Liu M. et al., 2020; Wilson et al., 2020; Xiang et al., 2020; Xiang et al., 2021; Gherardelli et al., 2022; Lu et al., 2022; Wiseman et al., 2023). Similarly, in transgenic mice overexpressing pathogenic mutant tau, lithium therapy inhibited GSK-3 activity and tau phosphorylation (Noble et al., 2005; Engel et al., 2006; Caccamo et al., 2007; Leroy et al., 2010; Shimada et al., 2012). However, the exact mechanisms of lithium’s neuroprotective properties in the context of AD remain unclear. The neuroprotective effects of lithium may be attributed to the inhibition of GSK-3β and IMPase, as well as the regulation of subsequent cascade reactions (Forlenza et al., 2014).

### 3.1 What is GSK-3 and why is it important?

GSK-3 is a serine/threonine-protein kinase that plays a crucial role in various physiological processes (Lauretti et al., 2020). There are two mammalian isoforms of GSK-3, α and β, which share a highly conserved (98%) catalytic structural domain but differ in their terminal regions (Liu and Klein, 2018; Marosi et al., 2022). The β isoform is more abundant in the brain and its expression level increases with age (Grimes and Jope, 2001b; Lee et al., 2006). Abnormal increases in GSK-3β levels and activity in the brain are associated with the pathogenesis of AD (Avila et al., 2010). GSK-3 represents one of the primary pharmacodynamic targets of lithium, which inhibits GSK-3 activity through three distinct mechanisms. At first, lithium competes with Mg^2+^ ions to bind to the catalytic site of GSK-3, which is necessary for enzyme activation, thus inhibiting GSK-3 activity (Ryves and Harwood, 2001; Ryves et al., 2002). Moreover, lithium enables the phosphorylation of specific serine residues, specifically serine-21 for GSK-3α and serine-9 for GSK-3β, within its regulatory amino-terminal domain by activating the phosphatidylinositol 3-kinase (PI 3-K)/serine/threonine kinase Akt-1 signaling pathway (Chalecka-Franaszek and Chuang, 1999; Zhang et al., 2003). Additionally, lithium inhibits the mRNA transcription of GSK-3, which reduces its expression level (Mendes et al., 2009).

Lithium has several neuroprotective mechanisms in AD related to GSK-3. In particular, lithium inhibits GSK-3β, which interferes with Wnt/β-catenin signaling pathway and modulates several downstream pathological processes (Sang et al., 2001; Sun et al., 2002; Ly et al., 2013). Research indicates that lithium inhibits the phosphorylation and subsequent degradation of β-catenin by inhibiting GSK-3β. This leads to the intracellular accumulation of β-catenin, which facilitates its entry into the nucleus where it interacts with T-cell factor/lymphoid enhancer-binding factor (TCF/LEF), regulating the transcription of target genes (Cadigan and Nusse, 1997; Toledo and Inestrosa, 2010). Ultimately, this reduces Aβ deposition, enhances neurogenesis, and improves mitochondrial bioenergetics (Lie et al., 2005; Fiorentini et al., 2010; Wilson et al., 2017; Martin et al., 2018). Furthermore, lithium regulates the activation of the nuclear factor erythroid 2-related factor (NRF2)/heme oxygenase-1 (HO-1) pathway through GSK-3β, which helps prevent oxidative damage (Chen et al., 2016). It also reduces neuroinflammation by inhibiting nuclear factor kappa-B (NF-κB) and signal transducer and activator of transcription 3 (STAT-3) (Beurel and Jope, 2008; Sakrajda and Szczepankiewicz, 2021). These effects are essential for maintaining nervous system health and play a significant role in the treatment of AD.

### 3.2 Lithium suppresses amyloid-beta pathology

**FIGURE 3:**
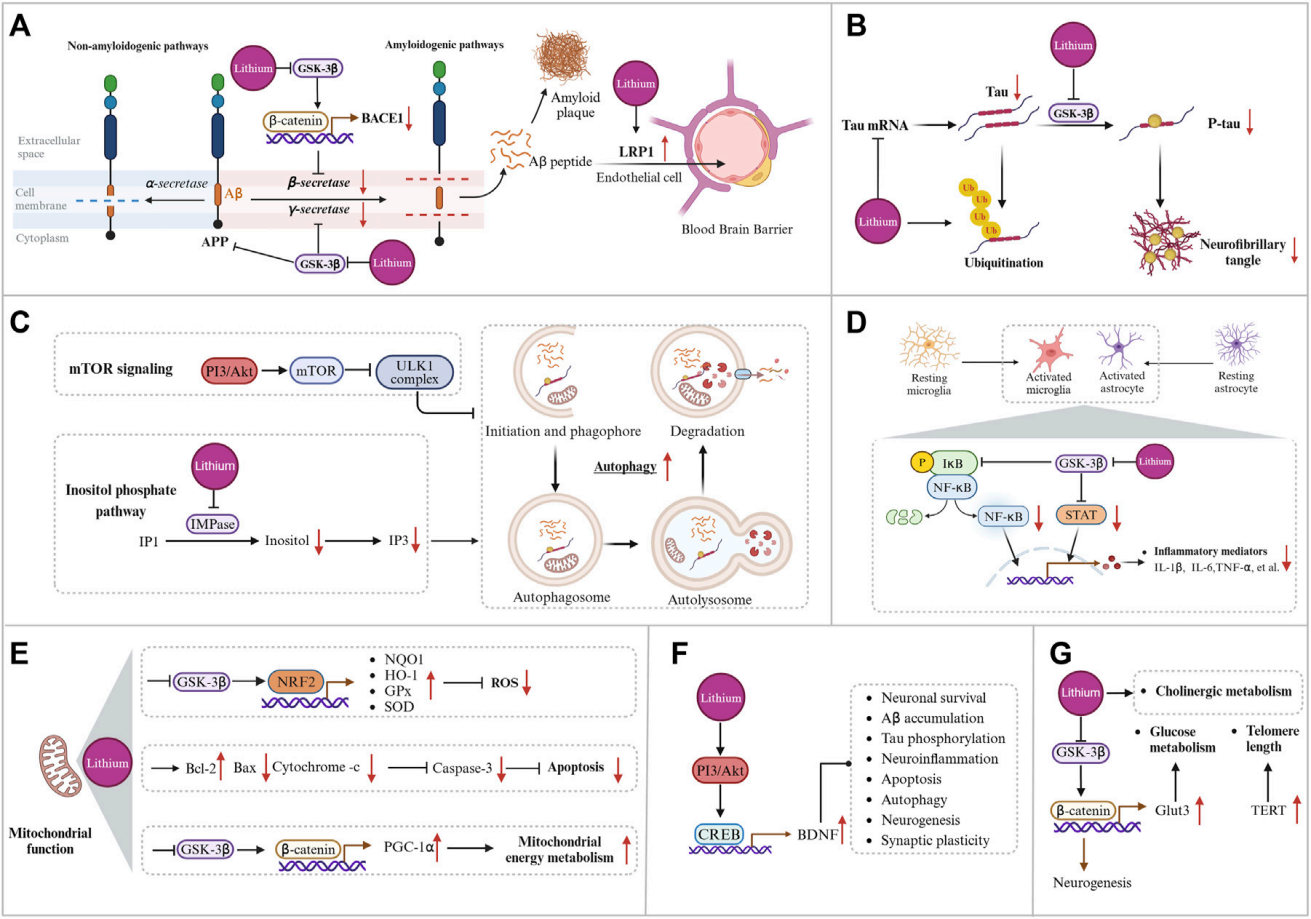
Potential mechanisms of lithium treatment for Alzheimer’s disease. **(A)** Lithium modulates the production and clearance of Aβ by inhibiting BACE-1 expression, reducing APP phosphorylation levels, decreasing γ-secretase activity, upregulating the BBB microvessel efflux transporter LRP1, and increasing CSF bulk-flow. **(B)** Lithium reduces total tau levels by decreasing tau mRNA levels and promoting tau ubiquitination, and reduces tau phosphorylation levels by inhibiting GSK-3β. **(C)** Lithium enhances autophagy through the phosphatidylinositol signaling pathway. **(D)** Mechanisms of lithium regulation of inflammation. **(E)** Mechanism of lithium regulating mitochondrial function. **(F)** Lithium affects downstream pathological events by promoting BDNF transcription. **(G)** Lithium regulates glucose metabolism, cholinergic metabolism, and telomere length. Created with BioRender.com.

According to the amyloid cascade hypothesis, abnormal deposition of Aβ is a crucial step leading to neuronal loss and death. Amyloid precursor protein (APP) can produce several bioactive fragments via both amyloidogenic and non-amyloidogenic pathways (Müller et al., 2017). In the amyloidogenic pathway, APP undergoes sequential cleavage by β-secretase (BACE1, β-site APP cleaving enzyme 1) and γ-secretase to produce Aβ. Aβ is released extracellularly, with Aβ_1-42_ being the most toxic form. Aβ can aggregate from monomeric forms into oligomers, protofibrils, and plaques, ultimately leading to neuronal death. In the non-amyloidogenic pathway, APP is initially cleaved by α-secretase into the non-toxic APP-derived C-terminal fragments (APP-CTFs), which are subsequently cleaved by γ-secretase into P3 and the APP intracellular domain (AICD), neither of which is associated with AD pathology (O'Brien and Wong, 2011). The amyloid and the non-amyloid pathways compete, and increased α-secretase activity significantly reduces Aβ production. Mutations in the *APP*, *BACE1*, presenilin 1 (*PS1*), and presenilin 2 (*PS2*) genes can affect Aβ production and aggregation in AD (Haass et al., 2012). Research suggests that lithium can reduce Aβ accumulation in the brain through various mechanisms, such as inhibiting Aβ synthesis and enhancing Aβ efflux across the blood-brain barrier (BBB) (Rockenstein et al., 2007; Sofola-Adesakin et al., 2014; Pan et al., 2018). Studies on transgenic animal models of AD indicate that lithium therapy can reduce Aβ_1-42_ levels and decrease the size and number of senile plaques in brain regions (Table 1). Figure 3A illustrates the mechanism by which lithium regulates Aβ.

BACE1 is essential for producing monomeric forms of Aβ that can aggregate and potentially trigger AD toxicity (Hampel et al., 2021). Therefore, BACE1 is a significant drug target for slowing early Aβ production in AD (Vassar, 2002). The Wnt/β-catenin pathway regulates the expression of BACE1 (Jia et al., 2019). Lithium inhibits GSK-3β activity, increasing nuclear β-catenin levels and activating the Wnt/β-catenin pathway. The translocation of β-catenin into the nucleus leads to its interaction with TCF4, which binds to the *BACE1* promoter. This results in the inhibition of *BACE1* transcription and ultimately leads to decreased Aβ production (Parr et al., 2015; Wilson et al., 2017). In addition, lithium can block the production of Aβ by interfering with the cleavage of APP by γ-secretase, while leaving Notch processing unaffected (Phiel et al., 2003). Specifically, in human brain and mammalian cells, PS1, the catalytic subunit of the γ-secretase complex, has mutations in AD brains that increase its binding capacity to GSK-3β (Takashima et al., 1998). Lithium can inhibit GSK-3β and decrease γ-secretase activity, which can interfere with APP cleavage and result in reduced Aβ production (Su et al., 2004). To summarize, lithium can regulate the production of Aβ by interfering with β- and γ-secretase during APP cleavage mediated by GSK-3β. Furthermore, GSK-3β is involved in the phosphorylation and maturation of APP, crucial processes for Aβ production (Zhang et al., 2019). The phosphorylation of APP by GSK-3β promotes its cleavage by β- and γ-secretase, leading to an increase in Aβ production (Aplin et al., 1996). Lithium, by inhibiting GSK-3β, reduces the phosphorylation level of APP, which in turn decreases Aβ production (Rockenstein et al., 2007). Additionally, a quantitative proteomics study showed that lithium’s inhibition of GSK-3 led to alterations in membrane proteins that are involved in APP processing. These changes included a reduction in lamin B1 and lamin B receptor, as well as an increase in several endosome-regulating rab proteins (rab5, rab7, and rab11) (Thompson et al., 2009). This suggests an alternative regulatory pathway for Aβ production.

Enhancing the efflux of Aβ across the BBB represents a potential therapeutic strategy. The BBB is a unique structure in the central nervous system (CNS) composed of brain microvascular endothelial cells, pericytes, astrocytes, and basement membranes. It is critical for molecular exchange and maintaining the relative stability of the brain’s internal environment (Zlokovic, 2008). At the BBB, Aβ clearance is facilitated by Aβ-trafficking proteins, such as P-glycoprotein and low-density lipoprotein receptor-related protein-1 (LRP1), which are essential for maintaining low levels of Aβ in the healthy brain (Cirrito et al., 2005; Storck et al., 2016). The administration of lithium leads to the upregulation of the BBB microvessel efflux transporter LRP1 and an increase in cerebrospinal fluid (CSF) bulk-flow. This enhancement promotes the clearance of Aβ by facilitating Aβ efflux and improves cognitive function in mouse models (Pan et al., 2018). Therefore, lithium has the potential to be a valuable therapeutic agent for AD as it impacts multiple stages of Aβ production and clearance.

### 3.3 Lithium suppresses tau pathology

Tau is a microtubule-binding protein that stabilizes axonal structures and participates in axonal nutrient transport and signaling (Baker and Götz, 2016). Most tau isoforms undergo post-translational modifications, primarily phosphorylation and dephosphorylation (Li and Götz, 2017; Lauretti and Praticò, 2020). Dephosphorylated tau promotes microtubule assembly, which is essential for cytoskeletal stability and the maintenance of healthy neuronal structure and function (Gong et al., 2005). Maintaining a balance between tau phosphorylation and dephosphorylation is critical for neuronal homeostasis under normal physiological conditions (Martin et al., 2013). In AD, tau hyperphosphorylation leads to the formation of intra-neuronal paired helical filaments and NFTs, which disrupt normal axonal messaging and ultimately cause cell death (Gendron and Petrucelli, 2009; Rudrabhatla et al., 2011). Several protein kinases, such as calmodulin-dependent protein kinase II, cyclic adenosine monophosphate (cAMP)-dependent protein kinase (PKA), GSK-3β, and cyclin-dependent protein kinase 5, can phosphorylate tau (Wang et al., 2007). Protein phosphatase-2A is considered the primary dephosphorylating enzyme for P-tau (Chang et al., 2018). Preclinical research indicates that lithium primarily attenuates tau phosphorylation by inhibiting GSK-3β (Table 1).

In primary neuronal cultures, lithium treatment reduces tau phosphorylation, enhances tau binding to microtubules, and facilitates microtubule assembly by inhibiting GSK-3 (Hong et al., 1997; Muñoz-Montaño et al., 1997; Lovestone et al., 1999; Takahashi et al., 1999). These results are consistent with *in vivo* studies. Treatment with lithium in transgenic mice overexpressing mutant human tau significantly inhibited GSK-3 activity, reduced tau phosphorylation, and significantly decreased the levels of aggregated insoluble tau (Pérez et al., 2003; Noble et al., 2005; Shimada et al., 2012). Long-term low-dose lithium treatment has been found to reduce P-tau levels and alleviate cognitive impairment in APP/PS1 transgenic mice (Liu M. et al., 2020). In a 12-month double-blind trial of lithium therapy, targeting serum lithium levels of 0.25–0.5 mmol/L, there was a significant decrease in cerebrospinal fluid P-tau concentrations (Forlenza et al., 2011). Although these findings support the potential of lithium to suppress tau pathology, several studies have reported inconsistent findings. In a 10-week multicenter randomized single-blind placebo-controlled trial, lithium therapy was administered with target serum levels of 0.5–0.8 mmol/L. The results showed no significant effects on cerebrospinal fluid Aβ, P-tau concentrations, or cognitive performance (Hampel et al., 2009). Variability in clinical trial results may be due to differences in the timing of administration and target serum concentrations. Furthermore, research has shown that extended lithium administration in mice overexpressing FTDP-17 tau and GSK-3β inhibited the hyperphosphorylation of tau and the formation of NFTs. However, it was unable to reverse pre-existing NFTs (Engel et al., 2006). This indicates that early intervention with lithium may impede the advancement of tau pathology in AD.

In addition, reducing total tau levels is being considered as a potential therapeutic approach for AD. It has been reported that lithium may modulate tau expression at the mRNA level. Cortical neuronal cultures exposed to lithium exhibited reduced levels of tau mRNA and protein expression (Rametti et al., 2008; Martin et al., 2009). In a mouse model of tauopathies, chronic lithium administration was observed to attenuate tau pathology by increasing tau ubiquitination, instead of suppressing tau phosphorylation (Nakashima et al., 2005). These findings propose an alternative mechanism through which lithium modulates tau pathology, separate from the GSK3β-mediated inhibition of tau phosphorylation. However, prolonged inhibition of tau may present challenges. Tau typically collaborates with APP to transport iron to the neuronal surface and promote iron efflux, thereby reducing neuronal iron levels (Lei et al., 2012). Prolonged inhibition of soluble tau results in the accumulation of iron in neurons to toxic levels, leading to a range of diseases related to iron deposition (Lei et al., 2017). Therefore, modest reductions in tau levels may be a more viable therapeutic strategy. Figure 3B illustrates the mechanism by which lithium modulates tau pathology.

### 3.4 Lithium activates autophagy

Autophagy is a cellular process in eukaryotic cells that degrades long-lived proteins, misfolded proteins, and damaged organelles (Klionsky and Emr, 2000). There are three primary types of autophagy: chaperone-mediated autophagy, microautophagy, and macroautophagy. Macroautophagy, also known as autophagy, is the most extensively studied and highly dynamic form of autophagy in eukaryotic cells (Zhang et al., 2021). Figure 3C illustrates the four stages of autophagy, including initiation and phagophore, autophagosome formation, autolysosome formation, and degradation (Kiriyama and Nochi, 2015). Each stage is tightly regulated. Autophagy plays an important role in AD pathogenesis by facilitating the degradation of Aβ, P-tau, and damaged mitochondria (Zhang et al., 2023). The study identified an accumulation of autophagic vacuoles in certain neuronal regions of the brain affected by AD, suggesting a potential impairment of the autophagic process (Boland et al., 2008). Another study demonstrated that enhancing mitochondrial autophagy improved cognitive function in AD (Fang et al., 2019). Therefore, enhancing autophagy may be a critical strategy for treating AD. The mammalian target of rapamycin (mTOR) is a well-known inhibitor of autophagy. It works by inhibiting the activity of the ULK1 complex, which is hyperactivated in MCI/AD brains and associated with the accumulation of Aβ and P-tau. Inhibition of mTOR signaling can activate autophagy and improve cognitive function in AD by reducing Aβ and P-tau deposition (Zhang et al., 2021).

Lithium enhances autophagic substrate clearance through mTOR-independent pathways (Sarkar et al., 2005). Specifically, IMPase catalyzes the hydrolysis of inositol monophosphate (IP1) to produce free inositol, which is necessary for the phosphatidylinositol signaling pathway (Sarkar and Rubinsztein, 2006). The autophagy-enhancing effects of lithium are a result of inhibiting IMPase and inositol polyphosphate-1-phosphatase (IPPase), which leads to a depletion of free inositol and decreased levels of inositol 1,4,5-trisphosphate (IP3) and diacylglycerol (DAG) (Serretti et al., 2009; Motoi et al., 2014). This autophagic effect of lithium promotes the clearance of aggregation-prone proteins and has potential for treating neuropsychiatric disorders (Damri et al., 2020). Inhibition of GSK-3β may actually decrease autophagy through the activation of the mTOR pathway (Sarkar et al., 2008). Notably, inhibition of the phosphatidylinositol signaling pathway by lithium and inhibition of the mTOR pathway by rapamycin, can work together to activate autophagy. Therefore, combination therapy with lithium and rapamycin has the potential to enhance the clearance of mutant aggregation-prone proteins and provide protection against their aggregation and toxicity (Sarkar and Rubinsztein, 2006). However, further confirmation of this mechanism in the context of AD is necessary.

### 3.5 Lithium suppresses inflammation

Neuroinflammation is a crucial factor in the development of AD. Evidence suggests that the overactivation of microglia, and astrocytes, as well as the inflammatory molecules they produce, can disrupt the neuronal microenvironment and lead to cognitive impairment (Uddin et al., 2020). Neuroinflammation is a double-edged sword for the brain. On one hand, it activates the immune system by initiating phagocytosis by glial cells to eliminate potential pathogens, which is beneficial. On the other hand, overactivation of neuroinflammation can lead to detrimental effects on neurons (Triviño and Von Bernhardi, 2021; Twarowski and Herbet, 2023). Microglia represent the primary immune cells responsible for maintaining homeostasis within the CNS (Bennett and Bennett, 2020). In response to immune stimulation or tissue injury, microglia undergo a morphological shift from a resting state of ramified morphology to an activated amoeboid morphology (Cai et al., 2022). Reactive microglia can be classified into two distinct categories, M1 pro-inflammatory and M2 anti-inflammatory, based on their role in neuroinflammation (Hristovska and Pascual, 2015). M1 microglia are activated by interferon-γ, tumor necrosis factor-alpha (TNF-α), or lipopolysaccharide and express pro-inflammatory cytokines such as interleukin-1β (IL-1β), IL-6, IL-18, and TNF-α, nitric oxide (NO), and reactive oxygen species (ROS), which are closely related to the inflammatory response and increased neurotoxicity (Block et al., 2007). M2 microglia are induced by IL-4 and IL-13 and are characterized by the production of anti-inflammatory cytokines (IL-4, IL-10, IL-13, and transforming growth factor-β), neurotrophic factors-1, and the promotion of phagocytosis of cellular debris and misfolded proteins and neuronal survival (Colton, 2009). In the early stages of AD, activated microglia are capable of phagocytosing and clearing Aβ and insoluble tau inclusion bodies (Takata et al., 2010; Brelstaff et al., 2018). However, as the disease progresses, elevated levels of proinflammatory cytokines induce a shift in microglia phenotype from M2 to M1, thereby reducing their phagocytic capacity (Tang and Le, 2016). In the presence of endogenous stimuli, such as Aβ and P-tau, which can interact with astrocytes to produce a proinflammatory phenotype and elevate proinflammatory levels, a sustained inflammatory response ultimately results in neuronal loss (Tang and Le, 2016). Consequently, the reduction of neuronal damage resulting from the inflammatory response represents a crucial aspect of AD treatment.

Lithium treatment attenuated the inflammatory responses of microglia and astrocytes and reduced the production of the pro-inflammatory factors IL-1β and TNF-α in the brains of AD animal models (Toledo and Inestrosa, 2010; Budni et al., 2017). In the McGill-R-Thy1-APP transgenic rat model, treatment with the new microdose lithium formulation, NP03, resulted in a reduction of neuroinflammatory markers. This included a decrease in chemokine (C-X-C motif) ligand 1 (CXCL1), IL-6, the expression of the microglial surface receptor Trem2, and microglial recruitment to Aβ-loaded neurons in the CA1 region of the hippocampus (Wilson et al., 2018; Wilson et al., 2020). In summary, while many studies suggest that lithium treatment reduces pro-inflammatory cytokine levels in AD models, the exact mechanism behind this modulation remains unclear. NF-κB, a pro-inflammatory transcription factor implicated in promoting neurodegeneration, is critical for innate immune responses. Its activation stimulates the transcription of pro-inflammatory genes and cytokine production (Sun et al., 2022). GSK-3β affects several transcription factors, including NF-κB (Steinbrecher et al., 2005). As a GSK-3β inhibitor, lithium reduces the transcriptional activity of NF-κB, leading to a decrease in the release of pro-inflammatory factors and glial cell activation (Yuskaitis and Jope, 2009; Sakrajda and Szczepankiewicz, 2021). Additionally, GSK-3β may regulate inflammation through STAT-3, which is also downregulated by lithium treatment, resulting in reduced secretion of pro-inflammatory cytokines (Beurel and Jope, 2008; Beurel and Jope, 2009). In summary, lithium’s inhibition of GSK-3β may regulate downstream inflammation through both the NF-κB and STAT-3 pathways. Figure 3D illustrates the mechanism by which lithium inhibits inflammation.

### 3.6 Lithium regulates mitochondrial function, oxidative stress, and apoptosis

Under physiological conditions, healthy mitochondria support neuronal activity by providing adequate energy to neurons and facilitating other related mitochondrial functions (Bélanger et al., 2011; Wang et al., 2020). Mitochondrial dysfunction in AD is characterized by decreased activities of mitochondrial complexes I (NADH: ubiquinone oxidoreductase), IV (cytochrome c oxidase), and V (adenosine triphosphate synthase), as well as the pyruvate and α-ketoglutarate dehydrogenase complexes. This is accompanied by an increased production of ROS (Johri, 2021). In AD, mitochondria are affected in both quantity and morphology (Tyumentsev et al., 2018). There is a correlation between reduced activity of enzymes involved in energy metabolism and cognitive performance in patients with AD (Pérez et al., 2017; Fišar et al., 2019). Mitochondrial dysfunction, and associated oxidative stress, cerebral metabolic failure, deregulation of calcium signaling, and cell death events (apoptosis) are all significant factors in the pathogenesis of AD (Perez Ortiz and Swerdlow, 2019; Ashleigh et al., 2023). While Aβ can disrupt mitochondrial function, leading to caspase-dependent neuronal apoptosis and the release of ROS (Zhang H. et al., 2012). Lithium has been found to play an important role in regulating mitochondrial function. The peroxisome proliferator-activated receptor gamma coactivator 1-α (PGC-1α) is a crucial regulator of mitochondrial function (Olson et al., 2008). Lithium may help maintain metabolic integrity by modulating NAD(P)H metabolism through GSK-3β inhibition. This enhances PGC-1α protein stability, nuclear localization, and transcriptional coactivation, elevating mitochondrial respiration and membrane potential (Martin et al., 2018). Figure 3E illustrates the mechanism by which lithium regulates mitochondrial function.

Additionally, dysfunction of mitochondria can lead to an increase in ROS production. Lithium benzoate can reduce ROS levels in cells, enhance spare respiratory capacity for the mitochondrial function, and protect against apoptosis (Lu et al., 2022). The cellular antioxidant network, which comprises catalase (CAT), superoxide dismutase (SOD), and glutathione peroxidase (GPx), plays an important role in scavenging ROS (Ashleigh et al., 2023). NRF2 is a crucial regulator of oxidative stress as it controls the expression of several genes, including NAD(P)H-quinone oxidoreductase 1 (*NQO1*), *HO-1*, and *GPx*, through the KEAP1-NRF2 pathway (Buendia et al., 2016; Sun et al., 2016). The regulation of NRF2 is dependent on GSK-3β (Chen et al., 2016). A study showed that lithium treatment decreased the protein levels of phospho-GSK3β (ser9), increased the protein levels of NRF2 and HO-1, enhanced the activity of SOD and GSH-Px, and reduced MDA levels in the brains of APP/PS1 mice compared to wild type mice (Xiang et al., 2020). The GSK-3β/NRF2/HO-1 pathway is primarily responsible for lithium’s ability to attenuate oxidative stress (Sotolongo et al., 2020; Xiang et al., 2020).

Apoptosis is a type of programmed cell death that occurs both physiologically and pathologically (Tuzlak et al., 2016). Failure of apoptosis may contribute to several forms of cancer, while excessive apoptosis is associated with neurodegenerative diseases, including AD (Kumari et al., 2023). The mitochondria-associated apoptotic process primarily involves an endogenous pathway with key factors such as cytochrome c and the pro- and anti-apoptotic proteins Bax and Bcl-2. The pro-apoptotic protein Bax initiates the permeabilization of the outer mitochondrial membrane, enabling cytochrome c to exit from the intermembrane space (Jourdain and Martinou, 2009). In the presence of adenosine triphosphate (ATP), cytochrome c binds to apoptotic protease activating factor-1 (Apaf-1) to form the Apaf-1/cytochrome c complex. The activation of procaspase-9 is initiated by this complex, leading to the formation of apoptotic bodies and the activation of caspase-9 and caspase-3 (Zou et al., 1999; Elena-Real et al., 2018). A study was conducted to investigate the neuroprotective effects of lithium against glutamate excitotoxicity. The study found that long-term treatment of cerebellar granule cells with lithium chloride resulted in decreased mRNA and protein levels of the pro-apoptotic Bax. Additionally, it significantly increased the mRNA and protein expression of the anti-apoptotic Bcl-2 (Chen and Chuang, 1999). In patients with bipolar disorder who are treated with lithium, there is an increase in the expression of Bcl-2 and a decrease in the expression of several pro-apoptotic protein, including Bcl-2-antagonist/killer 1 (BAK1) and Bcl-2-associated agonist of cell death (BAD), as detected in peripheral blood (Lowthert et al., 2012). However, a study found that chronic lithium treatment significantly improved memory deficits but did not modulate caspase-3 activity (Gelfo et al., 2017). Therefore, additional studies are necessary to clarify the mechanism by which lithium regulates apoptosis.

### 3.7 Neurotrophic and other effects of lithium

Brain-derived neurotrophic factor (BDNF) is critical for maintaining synaptic plasticity in learning and memory processes and has high expression levels in the cerebral cortex and hippocampus (Dwivedi, 2009). In postmortem AD samples, BDNF mRNA levels and protein expression were found to be lower in the cerebral cortex compared to controls (Phillips et al., 1991; Connor et al., 1997). Similar observations were also discovered in animal models of AD (Peng et al., 2009). Furthermore, patients with AD who had higher levels of BDNF in their serum showed a slower rate of cognitive decline (Laske et al., 2011). In a clinical pilot study, researchers observed significant increases in serum BDNF levels and notable reductions in Alzheimer’s Disease Assessment Scale - Cognitive Subscale (ADAS-Cog) scores were observed in AD patients treated with lithium compared to those receiving a placebo (Leyhe et al., 2009). In addition, chronic lithium treatment has been shown to increase BDNF expression in primary neuronal cultures and rat brains (Fukumoto et al., 2001; De-Paula et al., 2016). Lithium can activate Akt through PI3K phosphorylation, leading to the phosphorylation and activation of cAMP-responsive element binding protein (CREB), which ultimately increases BDNF expression (Meffre et al., 2015; Rosa and Fahnestock, 2015). In AD, depletion of BDNF is associated with tau phosphorylation, Aβ accumulation, neuroinflammation, autophagy, and apoptosis (Smith et al., 2014; Wang et al., 2019; Gao et al., 2022). BDNF and its receptor, TrkB, are involved in neuronal survival, neurogenesis, and synaptic plasticity (Grimes and Jope, 2001a; Huang and Reichardt, 2001; Hashimoto et al., 2002; Lu et al., 2013).

Other mechanisms contribute to the neuroprotective effects of lithium therapy, including glucose metabolism, cholinergic hypothesis, the synaptic plasticity, neurogenesis, and telomere length. Treatment of APP/PS1 mouse models with lithium carbonate stimulates neuronal glucose uptake and replenishes ATP levels by preferentially oxidizing glucose via glycolysis. The mechanism involves the upregulation of glucose transporter 3 (Glut3), a major carrier of glucose uptake in neurons, and the activation of adenosine 5′-monophosphate (AMP)-activated protein kinase (AMPK) (Gherardelli et al., 2022). Additionally, the ‘cholinergic hypothesis’ plays a significant role in the pathogenesis of AD, with the progressive loss of cholinergic innervation contributing to cognitive decline (Hampel et al., 2018). Neuronal nicotinic acetylcholine receptors (nAChRs) play a critical role in cognitive, learning, and memory processes. In AD, the expression of nAChRs is decreased (Lombardo and Maskos, 2015). Treatment with lithium has been shown to alleviate impaired learning and memory function in APP/PS1 mice by promoting the expression of α7 nAChRs (Xiang et al., 2021). Moreover, lithium therapy can affect cholinergic metabolism by inducing proteasomal degradation of overexpressed acetylcholinesterase and modulating cholinesterase activity, thereby exerting neuroprotective effects (Jing et al., 2013; Zanandrea et al., 2018).

Neurogenesis is the process through which neural stem cells generate new neurons. Its dysregulation may be associated with cognitive deficits related to AD (Babcock et al., 2021). Studies indicate that lithium treatment may enhance neurogenesis in the adult hippocampus (Chen et al., 2000; Fiorentini et al., 2010; Wilson et al., 2017). This enhancement may be associated with the inhibition of GSK-3β and activation of the Wnt/β-catenin pathway (Contestabile et al., 2013). There is also an association between hippocampal synaptic plasticity and memory and cognitive function (Cuestas Torres and Cardenas, 2020). Research has shown that microdose lithium treatment can improve learning and memory in McGill R-Thy1-APP transgenic rats by restoring CREB regulated transcription coactivator 1 (CRTC1) promoter occupancy within synaptic plasticity genes, thereby increasing hippocampal synaptic plasticity (Wilson et al., 2017). In addition, telomere length is a biomarker of cellular senescence, and telomere shortening is implicated in age-related diseases, including AD (Scarabino et al., 2017). Chronic lithium treatment has been shown to increase telomere length in the brains of the triple-transgenic (3×Tg-AD) mouse model (Cardillo et al., 2018). Telomerase plays a critical role in maintaining telomere length and ensuring genomic integrity (Shim et al., 2021). Telomerase reverse transcriptase (TERT) is the catalytic subunit within the telomerase complex that regulates and limits telomerase activity (Zhang Y. et al., 2012). According to recent studies, lithium activates the Wnt/β-catenin pathway by inhibiting GSK-3β, which subsequently activates *TERT* transcription. This activation may affect telomere elongation (Zhang Y. et al., 2012). Further research is needed to fully understand these mechanisms.

## 4 Clinical studies of lithium treatment in Alzheimer's disease

Preclinical studies support the protective effects of lithium in AD, and have elucidated its mechanism of action, providing favorable support for clinical trials. Significant progress has been achieved in clinical research on lithium in AD, including observational studies and clinical trials. Lithium therapy has been shown to reduce the incidence of dementia in patients with bipolar disorder (Nunes et al., 2007; Kessing et al., 2010; Velosa et al., 2020). A study discovered that patients with AD and MCI had significantly lower total serum lithium levels compared to healthy controls (González-Domínguez et al., 2014). An epidemiological study, which used a Japanese national database, suggested that elevated lithium levels in drinking water may be associated with reduced AD prevalence in women, but not in men (Muronaga et al., 2022). Research from the United States demonstrated a negative correlation between trace lithium levels in water and AD mortality (Fajardo et al., 2018). The observational studies indicate a possible link between lithium and cognitive function in patients with AD.

Several clinical trials have shown beneficial effects of lithium supplementation in the treatment of AD (Matsunaga et al., 2015). Although results have been inconsistent, it is worth noting that lithium has shown promise in treating AD. In a 10-week randomized controlled trial, AD patients treated with lithium showed a significant reduction in ADAS-Cog scores and a significant increase in serum BDNF levels compared to those who received a placebo (Leyhe et al., 2009). Two separate randomized clinical trials conducted on patients with amnestic MCI (aMCI) have shown that chronic lithium treatment (with serum levels of 0.25–0.5 mmol/L) can reduce cognitive decline. Additionally, it can decrease CSF levels of P-tau and increase CSF levels of Aβ_1-42_ (Forlenza et al., 2011; Forlenza et al., 2019). A longitudinal analysis of cognitive and functional status conducted recently, 13 years after enrollment in a trial of lithium therapy, revealed that older adults with aMCI who were treated with lithium had better long-term cognitive outcomes than a matched sample without treatment (Damiano et al., 2023). The study suggests that lithium treatment may provide long-term and sustained neuroprotective benefits. However, a clinical trial evaluated the effects of short-term lithium treatment in patients with mild AD and found that the intervention did not improve cognitive performance or reduce GSK-3 activity and P-tau expression, despite achieving target serum lithium levels of 0.5–0.8 mmol/L (Hampel et al., 2009). Patients with AD often present with psychiatric symptoms such as agitation and aggression, which can be challenging to treat and distressing for both patients and caregivers (Zhao et al., 2016; Devanand, 2023). Lithium, a medication commonly used to treat bipolar disorder, and the combination of lithium with AD medications may improve cognitive function and behavioral symptoms in patients with AD. A case series involving three agitated patients with AD showed that lithium administration (initiating at 150 mg and increasing to 300 mg daily after 2 weeks) significantly reduced agitation symptoms, although with limited efficacy in improving cognitive function (Devanand et al., 2017). Conversely, some studies have suggested that low-dose lithium treatment is ineffective in treating agitation, but correlates with overall clinical improvement and a remarkable safety profile (Devanand et al., 2022). The variability in results may be due to the timing and dosage of lithium administration, and more extensive studies are needed to elucidate the effects of the lithium. Table 2 summarizes completed and ongoing clinical trials of lithium for the treatment of AD.

**TABLE 2:** Summary of clinical trials of lithium in Alzheimer’s disease.

Drug	Study design	Subject number	Length of study	Lithium level/dosage	Type of dementia	Outcome	References/identifier no
Li_2_CO_3_	Single-blind, cross-sectional	36	13 years after a clinical trial	0.2–0.5 mmol/L	aMCI	Having a better long-term global cognitive outcome	Damiano et al. (2023)
Li_2_CO_3_	Double-blind, placebo-controlled	77	12 weeks	150–600 mg/day	AD with agitation	Did not have a significant effect on the treatment of agitation, but was associated with global clinical improvement and excellent safety	Devanand et al. (2022)
Li_2_CO_3_	Double-blind, placebo-controlled	61	24-month treatment period and 24-month follow-up period	0.25–0.5 mEq/L	aMCI	Attenuating cognitive functional decline (24-month), and increasing in CSF Aβ_1-42_ (36-month)	Forlenza et al. (2019)
Li_2_CO_3_	Double-blind, placebo-controlled	45	12months	0.25–0.5 mmol/L	aMCI	Decreasing CSF P-tau concentrations; having better performance of cognitive subscale in attention tasks	Forlenza et al. (2011)
Microdose lithium	Double-blind, placebo-controlled	113	15 months	300 μg/day	AD	Preventing cognitive loss; no kidney or thyroid dysfunction or any other organic disorder	Nunes et al. (2013)
Lithium sulfate	Single-blind, placebo-controlled	71	10 weeks	0.5–0.8 mmol/L	Mild AD	Did not have a significant effect on cognitive performance and reduction of P-tau	Hampel et al. (2009)
Lithium sulphate	Single-blind, placebo-controlled	27	10 weeks	0.5–0.8 mmol/L	Mild AD	Increasing serum BDNF levels and decreasing ADAS-Cog scores	Leyhe et al. (2009)
NanoLithium^®^ NP03 (lithium citrate)	Double-blind, placebo-controlled; followed by open-label trial period to evaluate clinical safety and efficacy	68	Double blind 12-week -period; open-label 36-week period	3 mL per day (1.8 mg/day)	Mild-to-severe AD	Ongoing studies	NCT05423522
AL001 (lithium salicylate)	Double-blind, placebo-controlled	72	A 14-day treatment period and a 42-day follow-up period	9 cohorts multiple ascending doses	Mild to moderate AD and healthy adult subjects	Ongoing studies	NCT05363293
Li_2_CO_3_	Double-blind, placebo-controlled	80	2 years	0.5–0.8 mEq/L	MCI due to AD	Ongoing studies	NCT03185208

Aβ, amyloid-beta; AD, Alzheimer’s disease; ADAS-Cog, Alzheimer’s Disease Assessment Scale - Cognitive Subscale; aMCI, amnestic mild cognitive impairment; BDNF, brain-derived neurotrophic factor; CSF, cerebrospinal fluid; Li_2_CO_3_, lithium carbonate.

Nanolithium is an experimental product that encapsulates lithium citrate within Aonys, utilizing the innovative Aonys^®^ drug delivery technology. This encapsulation optimizes the bioavailability of the compound and reduces its toxicity by providing it with unique absorption and distribution characteristics (Mouri et al., 2014; Guilliot et al., 2024). After absorption through the oral mucosa, lithium nanoparticles attach to high-density lipoproteins (HDL) in the bloodstream, where they are transported as protected lipids. HDL then facilitates the intracellular release of lithium through lipoprotein receptors, particularly the scavenger receptor class B type I (SR-B1), which is widely expressed in the BBB (Saddar et al., 2013; Mouri et al., 2016). This cell-penetrating mechanism enables pharmacologically active concentrations of lithium to be effective at lower doses, reducing the toxic effects of higher doses (Guilliot et al., 2024). Research on animal models of AD indicates that treatment with nanolithium (40 μg Li/kg) inhibits GSK-3β, reduces BACE1 expression and activity, lowers amyloid levels, enhances hippocampal neurogenesis and synaptic plasticity, and improves memory function (Wilson et al., 2017). In addition, NP03 reduces markers of neuroinflammation and cellular oxidative stress and shows efficacy both pre- and post-Aβ plaque formation (Wilson et al., 2018; Wilson et al., 2020). The ongoing clinical trial for NanoLithium^®^ NP03 is a prospective, multi-center, randomized (1:1), placebo-controlled, double-blind, parallel-group study, followed by an open-label study, designed to evaluate the clinical safety and efficacy of NanoLithium^®^ NP03 in patients with mild to severe AD. Sixty-eight subjects were enrolled and randomized to receive either the study drug (NanoLithium^®^ NP03) or a placebo, with 34 subjects in each group. The study consisted of a 12-week double-blind phase followed by a 36-week open-label phase. Throughout the follow-up period, biomarkers, imaging assessments, and questionnaires will be used to evaluate the safety, efficacy, and potential for AD improvement of NanoLithium^®^ NP03. This study will provide additional insight into the treatment of AD patients with NanoLithium^®^ NP03 (ClinicalTrials.gov ID: NCT05423522).

AL001, an ionic cocrystal (ICC) consisting of lithium salicylate proline (LISPRO), represents a novel class of ICCs synthesized from lithium salts and organic anions through a crystal engineering approach. This compound retains the bioactivities of lithium and shows superior performance compared to lithium carbonate and lithium salicylate in preventing hippocampus-dependent associative memory decline and reducing irritability without affecting the body weight or internal organ growth of the mice (Habib et al., 2019). A Phase 1/2a multicenter, double-blind, randomized, placebo-controlled, multiple ascending dose (MAD) clinical trial is ongoing to evaluate the safety and maximum tolerated dose of AL001 in patients with mild to moderate AD and healthy adult subjects. Seventy-two participants were enrolled and randomized to receive either the study drug (active AL001) or a placebo. The study consisted of a 4-week screening period, a 14-day treatment period, and a 42-day follow-up period (ClinicalTrials.gov ID: NCT05363293). Another clinical trial investigating lithium is the LATTICE study. The study enrolled eighty MCI patients aged 60 years and older who were randomized to receive either lithium (lithium carbonate) or a placebo (oral capsule) for 2 years. Participants received annual neurocognitive assessments, measurements of AD biomarkers, and magnetic resonance imaging (MRI) scans to evaluate the effectiveness of lithium in preventing AD-related MCI and its impact on cognitive and brain changes in elderly patients (ClinicalTrials.gov ID: NCT03185208).

In summary, the available evidence supports the therapeutic potential of lithium in the treatment of AD. However, further clinical trials with larger sample sizes are needed to determine its effects conclusively. It is hoped that the three ongoing clinical trials will provide new insights into the treatment of AD with lithium. Recent meta-analyses support the efficacy of lithium in improving cognitive function in patients with MCI and AD (Matsunaga et al., 2015; Singulani et al., 2024). Furthermore, lithium may be more effective than aducanumab in improving cognitive function and low-dose lithium may be safer than aducanumab, lecanemab, donanemab in patients with AD (Terao et al., 2022; Terao and Kodama, 2024). These findings highlight the potential usefulness of lithium in the treatment of AD.

## 5 Potential side effects and safety concerns with lithium

**FIGURE 4:**
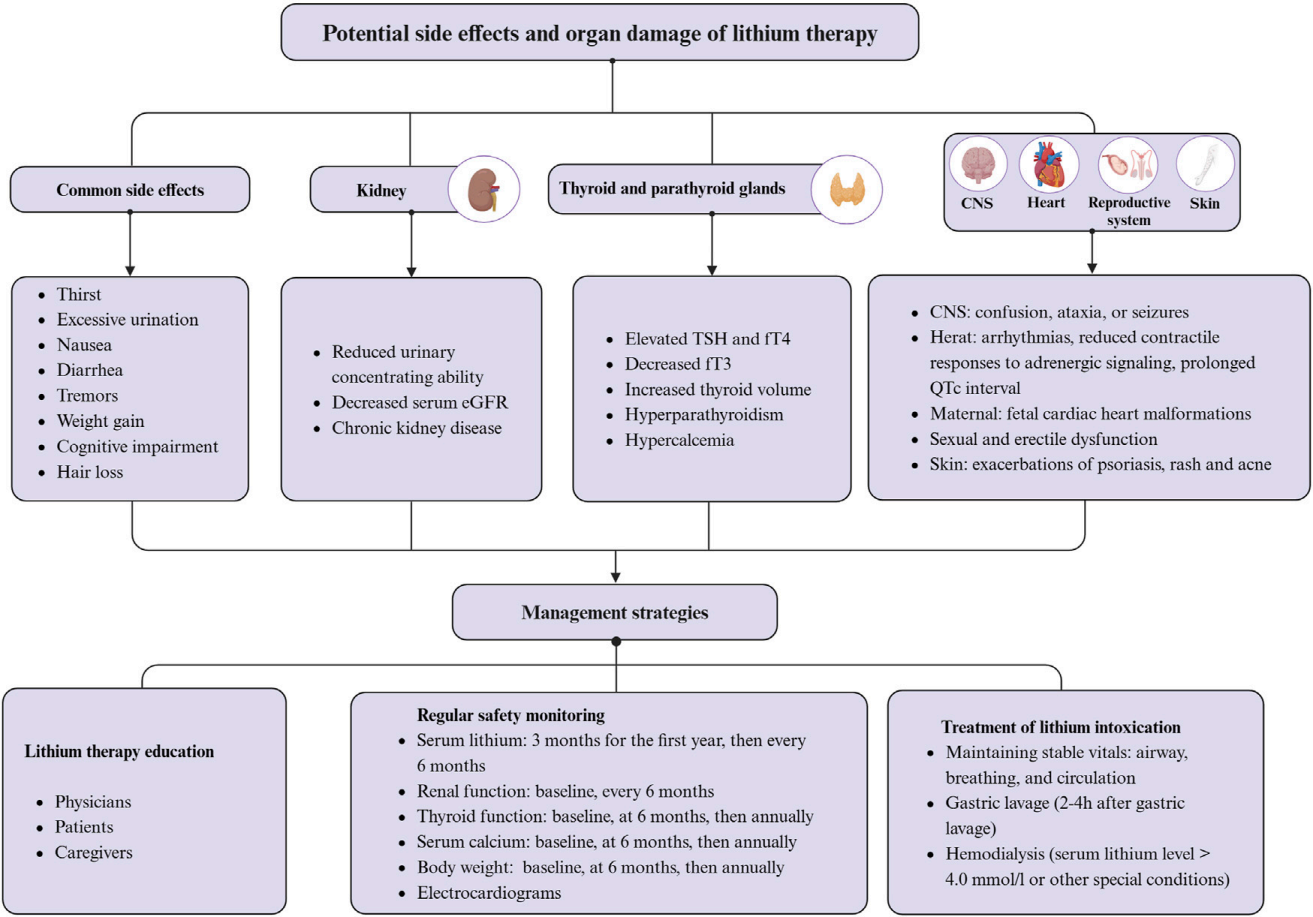
Potential side effects, organ damage, and management strategies associated with lithium therapy. Created with BioRender.com.

Lithium remains the most effective long-term treatment for bipolar disorder and shows considerable clinical promise for treating neurodegenerative diseases (Geddes et al., 2004; Chiu and Chuang, 2010). Despite its marked benefits, the use of lithium is associated with a number of potential side effects and safety concerns that require vigilance in clinical practice (McKnight et al., 2012). When taken orally, lithium is absorbed through the gastrointestinal tract and subsequently reaches brain concentrations that are approximately half of those in the serum. The kidneys primarily excrete lithium as free ions by the kidneys, and lithium clearance decreases with age. Therefore, renal function is critical for lithium metabolism and excretion (Grandjean and Aubry, 2009a). Consequently, any condition that affects circulating concentrations, such as dehydration, can lead to lithium toxicity. This toxicity usually reversible by dose reduction or discontinuation of treatment (Guilliot et al., 2023). However, prolonged high-dose lithium therapy can cause damage to the thyroid, parathyroid glands, and kidneys (Boivin et al., 2023). Figure 4 summarizes a summary of the potential side effects, organ damage, and management strategies associated with lithium therapy. The optimal plasma lithium concentration range for the treatment of bipolar disorder is relatively narrow, ideally between 0.6 and 1.2 mmol/L (Severus et al., 2008; Fountoulakis et al., 2017). Recent recommendations suggest a target serum lithium concentration range of 0.5–0.8 mmol/L for most patients with bipolar disorder, which is also the target concentration expected in AD clinical trials (Wijeratne and Draper, 2011). However, a lower therapeutic range of 0.5–0.6 mmol/L is generally recommended for elderly patients aged 50 years and older, and for those requiring concomitant medications that may interact with lithium in the presence of cardiac, renal, or thyroid disease (Wijeratne and Draper, 2011). Lithium therapy is associated with common side effects such as thirst, polyuria, nausea, diarrhea, tremor, weight gain, and cognitive impairment (Grandjean and Aubry, 2009b; Gitlin, 2016).

Symptoms of thirst and polyuria may be related to lithium’s inhibition of the G-protein-coupled pathway. Antidiuretic hormone activates this pathway, enhancing water reabsorption by aquaporin-2 (AQP2) in renal collecting duct cells. Lithium impairs renal concentrating ability by inhibiting the G-protein-coupled pathway and decreasing AQP2 expression, resulting in excessively diluted urine and indirectly inducing thirst (Marples et al., 1995). Tremor, primarily hand tremor, is one of the most common side effects of lithium, affecting approximately one quarter of patients, usually at the initiation or during dose adjustment, and decreasing over time (Gelenberg and Jefferson, 1995). Relief may be provided by reducing the dose of lithium, while beta-blockers, primaquine, and gabapentin may be used in patients with moderate to severe lithium-induced tremor (Baek et al., 2014). Severe tremor may be a sign of lithium toxicity and requires vigilance (Gelenberg and Jefferson, 1995). Nausea and/or diarrhea are also relatively common side effects of lithium treatment. Nausea affects 10%–20% of patients treated with lithium, typically early in treatment (Schou et al., 1970). Diarrhea affects approximately 10% of patients treated with lithium, with the incidence correlating with serum lithium levels above 0.8 mmol/L (Vestergaard et al., 1988). In addition, lithium administration resulted in weight gain of approximately 10 kg in 20% of patients, but the exact mechanism is unknown and may be related to increased intake of high-calorie beverages due to thirst (Vestergaard et al., 1980). Furthermore, about one in ten patients treated with lithium reported memory problems and poor concentration, although it is uncertain whether these effects are directly attributable to the drug (Vestergaard et al., 1980; Frisch et al., 2017). Lithium treatment may cause side effects such as exacerbation of psoriasis, nonspecific maculopapular rash, acne, and hair loss (Yassa, 1986; Yeung and Chan, 2004; McKnight et al., 2012). Proper education and monitoring can minimize these side effects in most cases. However, caution is advised in certain scenarios, such as pregnancy, even when the risk appears to be minimal (Oruch et al., 2014).

Long-term lithium treatment may increase the risk of chronic kidney disease, hyperthyroidism, hyperparathyroidism, and hypercalcemia (Czarnywojtek et al., 2020; Boivin et al., 2023). The risk associated with long-term lithium therapy may be twice that of non-lithium treatments and may increase with the duration of treatment (Van Alphen et al., 2021; Schoretsanitis et al., 2022). Therefore, it is necessary to monitor serum creatinine and estimated glomerular filtration rate (eGFR) at intervals of 6 months to 1 year during lithium therapy (Gitlin, 1993). Reduced lithium dose is recommended in patients with eGFR <60 mL/min (Boivin et al., 2023). Lithium has the potential to cause conditions such as goiter, hypothyroidism, or hyperthyroidism. The accumulation of lithium in the thyroid gland is 3–4 times higher than that in plasma (Czarnywojtek et al., 2020). Studies have shown significant correlations between long-term lithium treatment and several thyroid-related changes, including elevated TSH and fT4 levels, decreased fT3, increased thyroid volume, and nodular goiter, without affecting thyroid structure (Kraszewska et al., 2019). Therefore, it is essential to measure thyroid-stimulating hormone levels and thyroid ultrasound in patients on long-term lithium therapy before initiating treatment and then at 6- to 12-month intervals (Czarnywojtek et al., 2020). Additionally, lithium-induced hyperparathyroidism and hypercalcemia may result from its effects on the calcium-sensing receptor pathway and GSK-3 (Mifsud et al., 2020; Kovacs et al., 2022). Therefore, it is critical to regularly monitor calcium levels in patients on long-term lithium therapy (Mifsud et al., 2020). While lithium treatment may be linked to sexual and erectile dysfunction, the exact mechanisms behind this association remain unclear (Blay et al., 1982; Sheibani et al., 2022). According to research, indomethacin, aspirin, and sildenafil may be effective in treating sexual and erectile dysfunction caused by lithium (Gopalakrishnan et al., 2006; Sadeghipour et al., 2007; Saroukhani et al., 2013). Additionally, recent research indicates that serum lithium concentrations exceeding 1.5 mmol/L may have adverse effects on the myocardium and myocardial development in patients with bipolar disorder. This may result in arrhythmias, fetal cardiac malformations, and reduced contractile responses to adrenergic signaling (Mehta and Vannozzi, 2017; Patorno et al., 2017; Moradi et al., 2019; Hamstra et al., 2023). Animal studies have shown that treatment with lithium carbonate at a dose of 45 mg/kg for 12 weeks causes myocardial histopathologic damage (L'Abbate et al., 2023). Therefore, electrocardiograms should be monitored in patients undergoing long-term lithium therapy due to the associated prolonged QTc intervals (Mamiya et al., 2005).

Lithium overdose and toxicity can occur due to accidental or intentional consumption of excessive amounts of lithium, or increased lithium accumulation from ongoing chronic therapy (Oruch et al., 2014). Mild toxicity may result from lithium concentrations of 1.5–2.5 mmol/L, while moderate toxicity is associated with 2.5–3.5 mmol/L, and levels above 3.5 mmol/L can be fatal (MacLeod-Glover and Chuang, 2020). Recent data suggests that the mortality rate from lithium toxicity is less than 1% (Baird-Gunning et al., 2017). Symptoms of mild lithium toxicity include weakness, increased tremor, mild ataxia, poor concentration, and diarrhea. Symptoms such as vomiting, severe tremors, slurred speech, confusion, and drowsiness may occur when plasma levels exceed 1.5 mmol/L (Gadallah et al., 1988; MacLeod-Glover and Chuang, 2020). Lithium poisoning can be classified into acute, acute-on-chronic, and chronic forms. The latter two are more likely to involve the nervous system (Baird-Gunning et al., 2017; Hlaing et al., 2020). Clinical manifestations of CNS lithium toxicity range from asymptomatic to confusion, ataxia, and seizures (Baird-Gunning et al., 2017). Lithium toxicity can cause a chronic cerebellar disorder known as Syndrome of Irreversible Lithium-Effectuated Neurotoxicity (SILENT). This syndrome is characterized by persistent ataxia, nystagmus, and gait irregularities that last for more than 2 months after lithium exposure. Early recognition and intervention can prevent SILENT (Marmol et al., 2024). To prevent lithium toxicity, it is crucial to lower serum lithium concentrations, correct fluid and electrolyte imbalances, and prevent potential neurologic complications caused by the toxicity, especially in the elderly (Meltzer and Steinlauf, 2002). There is no antidote for lithium toxicity. To prevent side effects of lithium therapy, it is important to educate and assess various parameters such as serum lithium concentration, renal and thyroid function, serum calcium concentration, and body weight (Ng et al., 2009; Won and Kim, 2017). Education for physicians, patients, and caregivers, particularly for older adults taking lithium regularly, should include standardized administration of lithium salts and recognition of the signs and early symptoms of lithium toxicity (Schaub et al., 2001). Additionally, prompt management of lithium poisoning is essential, including maintenance of vital signs (airway, breathing, and circulation), and performing gastric lavage, and dialysis, if necessary (Haussmann et al., 2015).

Research on the role of lithium in AD therapy has primarily been limited to preclinical studies. Clinical trials are still in their early stages, and there is little research on potential side effects in AD. However, lessons learned from the use of lithium in studies of bipolar disorder may guide its future use in the treatment of AD (McKnight et al., 2012). A study on the feasibility and tolerability of low-dose lithium for the treatment of AD found that administering it to older patients resulted in fewer side effects compared to other treatments. These side effects were mild and resolved upon discontinuation of the drug, but the treatment was not completely safe (Macdonald et al., 2008). Clinical evidence indicates that low-dose lithium therapy (typically serum concentrations ≤0.5 mM) can improve cognitive function and behavioral symptoms in AD. These findings support the therapeutic value of low-dose lithium administration (Nunes et al., 2013; Mauer et al., 2014; Burhanullah and Rosenberg, 2022; Devanand et al., 2022; Hamstra et al., 2023). Therefore, low-dose lithium may reduce the side effects associated with higher doses. However, studies on the treatment of AD with trace lithium are currently limited. Further randomized controlled trials are needed to determine its therapeutic efficacy. Additionally, the identification of the optimal lithium salt form is essential for the development of long-term lithium therapies for AD. Novel lithium formulations, such as NanoLithium^®^ NP03 and AL001, which are in clinical trials, are expected to reduce side effects, improve safety, and show promise for AD therapy. Recent studies have also shown that the combining of the natural food Momordica charantia with lithium chloride reduces toxicity and improves cognitive function in patients with AD (Huang et al., 2018). Therefore, combination therapy may be an effective and safe approach to using lithium in AD treatment.

## 6 Conclusion and future

Lithium has been shown to have multiple protective effects in preclinical studies of AD. These effects include inhibiting GSK-3 activity, reducing Aβ deposition and tau phosphorylation, regulating autophagy, inflammation, oxidative stress, cholinergic and glucose metabolism, enhancing neurogenesis and synaptic plasticity, maintaining mitochondrial homeostasis, and improving cognitive function. Understanding these mechanisms is crucial to realizing the therapeutic potential of lithium and serves as the basis for its clinical application in the treatment of AD. The protective effects of lithium make it a promising candidate for AD treatment. Clinical studies have demonstrated that lithium treatment leads to significant cognitive improvements in AD patients compared to those who received a placebo. These preclinical and clinical studies provide preliminary support for the clinical use of lithium in AD. Although several studies support the efficacy of lithium in the treatment of AD, a few studies suggest that it may not be effective. This observed variability may be due to differences in lithium dosage and treatment duration between the studies. The efficacy of lithium in treating AD may be compromised if the dose administered is subtherapeutic or the duration of treatment is inadequate. Future studies should aim to determine the optimal drug concentration for lithium treatment of AD. Additionally, the reliability of the findings may be affected by the absence of large sample clinical trial data. Therefore, it is crucial to conduct larger multicenter clinical trials to validate the long-term effectiveness of lithium in treating AD.

Furthermore, while research suggests that lithium affects various therapeutic targets, the exact upstream molecular mechanisms have yet to be fully understood. Therefore, further in-depth research is needed to elucidate the molecular mechanisms and pathways by which lithium affects AD pathology. Additionally, long-term use of lithium may induce various side effects, such as impaired kidney function, thyroid and parathyroid abnormalities, and neurological damage. The balance between the side effects associated with lithium, safety concerns, and its clinical benefits is a significant issue for its clinical application. Lithium treatment has been associated with several side effects, most of which have been identified in patients with bipolar disorder. Research into its use in AD is still in its infancy. Appropriate lithium doses for AD are usually lower than those used for bipolar disorder. Although there is still a risk of side effects, it is expected to be lower. Regular monitoring can help mitigate this risk. It is anticipated that future research in lithium therapy will provide more effective and personalized therapeutic strategies for patients with AD, which can delay disease progression and improve quality of life.
